# Cultural uncertainty avoidance predicts consumers’ affective reactions to chemicals

**DOI:** 10.1111/risa.17693

**Published:** 2025-01-17

**Authors:** Christian Martin

**Affiliations:** ^1^ School of Business Maynooth University Maynooth Ireland

**Keywords:** affective reactions to chemicals, chemophobia, risk as feelings, societal context, uncertainty avoidance

## Abstract

Chemicals in general often evoke negative emotions (e.g., worry or fear) in consumers. This can cause consumers to avoid beneficial products and may even lead to suboptimal public policy decisions. It is, therefore, important to better understand how affective reactions to chemicals in general (ARC) form in order to be able to counteract these undesirable effects. The present research contributes to the literature on ARC by studying between‐country differences in ARC. While ARC were negative in all countries in our dataset, there were practically relevant between‐country differences in how negative they were. We predicted and found that consumers in higher uncertainty avoidance (UA) societies reported more negative ARC than their counterparts in lower UA societies. This effect was due to the rule orientation component rather than the anxiety component of UA. Importantly, while we found evidence for several alternative explanations for between‐country variation in ARC (i.e., differences in affluence, individualism, prevalence of chemicals, and trust in consumer protection), the UA effect remained statistically significant when we controlled for other country characteristics. The present research contributes to a better understanding of how contextual factors on the society level influence consumers’ ARC and in doing so advances our understanding of ARC. It also has implications for practitioners who wish to educate consumers on the risks and benefits of chemicals.

## 1 INTRODUCTION

Research suggests that consumers typically experience negative emotional reactions to chemicals (i.e., the concept of chemicals in general), such as worry or concern. Even strong emotional reactions, such as fear (i.e., chemophobia), seem to be common among consumers. This has been documented in a number of different studies over a longer period of time and in a range of different countries (Kraus et al., 1992; Rembischevski et al., 2022; Siegrist & Bearth, 2019). It has been suggested that consumers typically base decisions involving chemicals on their affective reactions rather than on information‐based cognitive processes (Bearth et al., 2019). However, consumers’ reliance on (negative) emotional reactions to chemicals rather than facts can result in them making suboptimal decisions or mishandling products (Saleh et al., 2019). For example, consumers may reject products that can help protect them, such as repellents or vaccines, purely based on the fact that they contain chemicals (Greenberg et al., 2020). Moreover, consumers widespread negative affective reactions to chemicals in general (ARC) may even lead to ill‐fated policy decisions or hinder technological progress (Siegrist & Bearth, 2019).

Given these undesirable consequences, it is not surprising that researchers have long sought to better understand why consumers tend to have negative ARC, such as fear or worry. Extant research has produced many important insights in this regard. However, so far, this research has predominantly focused on features of consumers and products and processes on the consumer level (Siegrist & Bearth, 2019). We contribute to this literature by studying how characteristics of the societal context in which consumers are embedded in shape their negative ARC. In particular, we propose that stronger cultural uncertainty avoidance (UA) will be associated with more negative ARC in consumers.

We study this effect in a dataset of over 27,000 consumers from 28 different countries. Our research sheds light on which component of UA (i.e., general anxiety or rule orientation) is responsible for the proposed effect, tests several alternative explanations for between‐country differences in ARC (i.e., differences in affluence, individualism, prevalence of chemicals, and trust in consumer protection), and uses instrumental variable regression to address endogeneity concerns (Antonakis et al., 2010). Our findings contribute to a better understanding of consumers’ ARC and why there are differences in ARC between consumer populations. In addition, our research has implications for practitioners who wish to educate consumers about the benefits and risks of chemicals.

### 1.1 Affective reactions to chemicals

Research suggests that consumers make sense of hazards and threats through cognitive (i.e., risk as analysis) and affective (i.e., risk as feelings) processes. Risk as analysis involves the processing of information, while risk as feelings describes affective reactions to mental imagery and associations related to hazards (Loewenstein et al., 2001; Slovic et al., 2004).

Cognitive and affective processes are also relevant when consumers judge chemical risks. Consumers may try to evaluate the risk of chemicals based on facts and their understanding of available information (Shim et al., 2011). It is often difficult to quantify the risk of a given chemical substance, however, and some uncertainty about the risk may remain, even among experts (Jansen et al., 2019; Kraus et al., 1992). Communication of chemical risks may, therefore, not be able to fully eliminate uncertainty about chemical risks for consumers (Jansen et al., 2020; Ueland et al., 2012). In addition, chemistry is a complex field of science that is often difficult to understand for laypeople (Hartings & Fahy, 2011). This makes it difficult to design communication that helps laypeople understand the results of risk assessments of chemicals (Bearth et al., 2017; Jansen et al., 2020).

Because of the difficulties related to interpreting and using available information, such as risk assessments, and because consumers often lack time, skills, and motivation to process available information, affective processes play an important role in how consumers perceive chemical risks (Bearth et al., 2019; Saleh et al., 2019).

Based on their intuition, consumers seem to have mostly negative reactions toward chemicals in general (Kraus et al., 1992). In a recent study, consumers frequently associated negatively connoted words with the concept of “chemical substances,” such as “poison,” “death,” or “dangerous” which in turn evoked negative affect (Saleh et al., 2019). This may explain why many consumers seem to fear chemicals (i.e., chemophobia) at least to some extent (Bearth et al., 2019; Rembischevski et al., 2022). Fear, thereby, is a fundamental negative emotion (Buckley, 2016; Watson et al., 1988) and is a typical affective reaction to hazards (Loewenstein et al., 2001). A fear of chemicals seems to be so widespread that scholars are worried about it negatively affecting their ability to communicate the benefits of chemicals and to introduce new innovative products, and that public policy may be based on this fear of chemicals rather than on facts and risk‐benefit assessments (Hartings & Fahy, 2011; Saleh et al., 2021; Siegrist & Bearth, 2019).

Consumers’ negative ARC seem to be rooted in their reliance on different heuristics (Siegrist & Bearth, 2019). One particularly relevant heuristic in this regard seems to be the “natural‐is‐better” heuristic (Siegrist & Hartmann, 2020). Consumers have a tendency to think that natural substances and products are preferable to their human‐made counterparts, because they perceive nature to be “safe and benevolent” (Scott & Rozin, 2020, p. 989). Important criteria for naturalness are thereby whether humans have changed a substance itself and/or whether something has been added to an otherwise natural substance (Scott & Rozin, 2017). Actions that compromise naturalness are viewed as risky (Sjöberg, 2000). The perception of naturalness is thereby particularly compromised when the changes or additions to a substance are chemical in nature (Rozin, 2005). Consumers then experience negative ARC because they seem to think of “synthetic chemicals” that have been generated by human tampering with nature when they think of “chemicals” (Saleh et al., 2019).

These negative ARC seem to vary in strength between countries. Recent research found between‐country differences in the strength of fear of chemicals in a set of eight European countries (Bearth et al., 2019). It is not well‐understood, however, why ARC vary between societies.

### 1.2 Uncertainty avoidance and affective reactions to chemicals

We propose that cultural UA can explain variation in between‐country differences in ARC. There are two prominent definitions of UA as cultural value in the literature. Hofstede (2011) defines UA as tolerance for ambiguity and the extent to which cultures employ rules and laws to avoid unstructured and unpredictable situations (Hofstede et al., 2010). Similarly, project GLOBE (2004b, p. 1) defines UA as the extent to which a society wishes to “rely on social norms, rules, and procedures to alleviate unpredictability of future events. The greater the desire to avoid uncertainty, the more people seek orderliness, consistency, structure, formal procedures, and laws to cover situations in their daily lives.” It is important to note that UA is conceptually distinct from risk aversion because of its focus on ambiguity and not risk (Hofstede et al., 2010; Minkov & Hofstede, 2014).

It has been suggested that UA influences consumers’ risk perception. This is because high UA cultures seek to avoid uncertainty because they seek to avoid potential failures and losses that may hide in uncertainty (Bontempo et al., 1997). For example, high UA cultures are willing to forgo experimentation and innovation for the sake of predictability and consistency (House et al., 2004). Because of this mindset, these cultures focus on potential losses when judging risks while discounting potential gains. This leads to a tendency to perceive hazards as more risky in higher compared to lower UA societies (Bontempo et al., 1997).

Evidence for this effect has been found in different contexts. For example, consumers from high UA cultures perceived the risk of infectious disease, terrorist attacks, and natural disasters at travel destinations to be greater than consumers from lower UA cultures (Kozak et al., 2007). There were similar differences in the perception of travel and destination risk in another study (Kim et al., 2016). Consumers also perceived the risk related to internet buying to be greater if they were from higher compared to lower UA cultures (Al Kailani & Kumar, 2011). Similarly, UA has been found to predict managers’ risk perception related to corporate takeovers (Frijns et al., 2013).

This effect is not limited to risk perceptions but also explains between‐country differences in risk as feelings. That is, consumers in higher UA cultures tend to report more negative affective reactions to hazards compared to consumers in lower UA societies. For example, a recent study suggests that higher UA is related to more negative affective reactions to the COVID pandemic (i.e., worries) and its impact on the economy and the health and safety of consumers (Rojas‐Méndez, 2021). In another study, consumers in higher UA countries reported stronger negative affective reactions to vaccines (i.e., concerns about potential side‐effects) than consumers in lower UA countries (Lu, 2023).

In line with these findings, we propose that UA will also predict consumers’ ARC. Theory suggests that consumers in higher UA societies tend to focus more on potential dangers compared to consumers in lower UA societies. This seems to translate into stronger negative affective reactions to hazards in higher (vs. lower) UA societies. As discussed in Section 1.1, most consumers’ intuition seems to be that chemicals are dangerous. Because of this, we predict that consumers in higher (lower) UA societies will experience stronger (less strong) negative ARC.

### 1.3 The present research

We tested our theoretical prediction using multilevel models. This type of model allowed us to test effects across different levels of analysis (Nezlek, 2008), such as how UA on the societal level relates to individual consumers’ ARC.

#### 1.3.1 Two operationalizations of UA

In this research, we used two different operationalizations of UA (i.e., Hofstede's and GLOBE's). While Hofstede's and GLOBE's definitions of UA are similar (see Section 1.2), they operationalize UA differently. Hofstede's measure is broader in scope and captures general anxiety^1^ and the extent to which societies are rule oriented. Anxiety is the more prominent UA component in Hofstede's index. GLOBE's measure, on the other hand, assesses only the extent to which cultures are rule oriented (Minkov & Hofstede, 2014; Venaik & Brewer, 2010). While some authors have argued that both operationalizations tap different constructs (de Mooij, 2015), others maintain that there is some overlap between the two different operationalizations of UA and that Hofstede's index is simply broader in scope and has a different focus than GLOBE's (Venaik & Brewer, 2010). We adopt the latter view based on a content analysis of the measurement tools (Venaik & Brewer, 2010).^2^

That both measures overlap to some extent but also differ from each other allowed us to test whether the rule orientation or the anxiety component of UA predicts ARC. Extant theorizing argues that consumers in higher UA societies wish to avoid failure rather than try to achieve success and, therefore, focus on potential losses while discounting potential gains. This difference between cultures can explain between‐country differences in risk perceptions (Bontempo et al., 1997) and affective reactions to hazards (see Section 1.2). Anxiety does not seem to play a role in that theoretical argument.

The cultural desire to avoid failures and losses is captured by the rule orientation component of UA. Rules are valued because they make situations more predictable (Minkov & Hofstede, 2014). In other words, rules are valued because they reduce the likelihood of failures and losses. Because of this, the extent to which cultures seek to avoid failures and losses can be measured by how much they are rule oriented. Hofstede and GLOBE both take this approach to measuring this aspect of UA (Venaik & Brewer, 2010). Accordingly, rule orientation, rather than anxiety, should explain between‐country differences in ARC. In line with this, we would expect that the Hofstede UA measure predicts little variation in ARC beyond GLOBE's, since GLOBE's index captures only rule orientation, while Hofstede's prominently features the anxiety component.

However, it is possible that national differences in anxiety contribute to explaining between‐country differences in ARC. This is because anxiety is associated with negative affective states (Watson et al., 1988). Hofstede's UA measure should predict ARC beyond GLOBE's if between‐country differences in ARC are indeed related to cultural differences in both UA components (i.e., rule orientation and anxiety).

#### 1.3.2 Alternative explanations

In our analyses, we considered several different alternative explanations for the proposed UA—ARC effect. First, it is possible that the UA—ARC effect is due to differences in the composition of consumer populations in different countries (Diez Roux, 2002). Different demographic variables have been found to be related to consumers’ risk judgments (Rowe & Wright, 2001) and ARC (Bearth et al., 2019; Nieto‐Villegas et al., 2023; Rembischevski et al., 2022). We account for the possibility of compositional effects by including individual‐level controls in our first‐stage model.

Second, it has been suggested that individualistic societies view innovative technologies more positively than less individualistic societies. This leads to more innovations in these countries, which in turn leads to more prosperity (Gorodnichenko & Roland, 2011). This would suggest that consumers in individualistic and affluent societies are less worried about innovative and novel technologies. More positive attitude toward innovation and technology may potentially be associated with less negative ARC. At the same time, consumers in individualistic countries have been found to perceive risk as being greater than consumers in more collectivistic countries. This is because in collectivistic countries, consumers’ social environment is more likely to offer support to consumers who experience significant losses, while in individualistic cultures, this may be less likely (Weber & Hsee, 1998). A similar argument can be made for less (vs. more) affluent societies. Taken together, affluence and individualism may be related to ARC even though extant research makes contradictory predictions regarding the sign of this relationship. At the same time, less affluent and less individualistic societies tend to score higher on UA (Minkov & Hofstede, 2014). Accordingly, differences in affluence and individualism might explain the UA—ARC relationship. We controlled for national affluence (i.e., GDP) and individualism to rule out this possibility.

The spatial proximity to a hazard has also been found to predict affective reactions, such as concern. Consumers, in many cases, are more concerned about a hazard that is spatially closer (vs. further away) from them (Hannon, 1994). However, there is also research that suggests that consumers are sometimes less concerned about a hazard that is closer to them. This has been termed the psychological typhoon eye effect (Li et al., 2009). These effects may translate to the country level and to ARC. The extent to which chemicals are prevalent in a given country may influence how concerned consumers are about them. We account for this possibility by including the relative importance of the chemical industry in a country and the amount of chemical waste produced in a country as controls in our models.

Lastly, trust in public authorities which regulate a given hazard has been found to reduce consumer's risk perception of that hazard (Siegrist, 2021). Consumers who trust authorities more also seem to have less negative ARC, such as fear, at least in some countries (Bearth et al., 2019; Saleh et al., 2019). We, therefore, controlled for trust in consumer protection to test whether UA effects may be due to differences in this type of trust between countries.

#### 1.3.3 Instrumental variable analysis

While we have carefully reviewed the literature to identify alternative explanations, it is possible that there are alternative explanations that we have not identified. We address this concern by using instrumental variable regression (see Sections 2.3 and D in the Supplementary Materials). This technique allowed us to estimate an unbiased UA effect that is robust to omitted variables (Angrist & Pischke, 2009; Antonakis et al., 2010).

## 2 METHOD

### 2.1 Dataset

Individual‐level data was taken from the Eurobarometer 92.4 dataset, version 1.0.0 (European Commission, Brussels, 2020). The Eurobarometer 92.4 was a multipurpose survey carried out in 2019 which collected information on a range of different topics. The dataset contains information on ARC for 27,042 participants from 28 countries. All participants for whom information on ARC, respectively, ARC and all individual‐level control variables, was available were included in our analyses. Information on the included countries and sample sizes can be found in Table 1. Individual‐level data was combined with information on different country‐level characteristics from other sources. We have always included all countries in a given statistical model for which information on all relevant country‐level indicator(s) was available.

**TABLE 1:** Information on countries in the dataset.

Country	ISO	*N* ^a^	ARC
Austria	AUT	1001	3.17
Belgium	BEL	1000	3.23
Bulgaria	BGR	981	3.41
Croatia	HRV	1020	3.45
Cyprus	CYP	501	3.81
Czech Republic	CZE	987	3.27
Denmark	DNK	1007	3.3
Estonia	EST	959	3.16
Finland	FIN	986	3.07
France	FRA	1011	3.4
Germany	DEU	1508	3.23
Greece	GRC	1005	3.6
Hungary	HUN	1025	3.25
Ireland	IRL	998	3.32
Italy	ITA	1004	3.28
Latvia	LVA	974	3.48
Lithuania	LTU	978	3.29
Luxembourg	LUX	502	3.42
Malta	MLT	487	3.53
Netherlands	NLD	1026	3.23
Poland	POL	1018	3.27
Portugal	PRT	979	3.43
Romania	ROU	1057	3.3
Slovakia	SVK	1012	3.32
Slovenia	SVN	999	3.47
Spain	ESP	1010	3.54
Sweden	SWE	1007	3.19
United Kingdom	GBR	1000	3.39

*Note*: ISO = country code; ARC = affective reactions to chemicals (i.e., estimated using Equation 1 without individual‐level control variables).

^a^ Only participants for which data on ARC was available were included.

### 2.2 Measures

#### 2.2.1 Affective reactions to chemicals

Consumers’ ARC were measured using two items in the Eurobarometer 92.4 questionnaire (i.e., “You are worried about the impact of chemicals present in everyday products on your health” and “You are worried about the impact of chemicals present in everyday products on the environment”). Participants responded to the statements on a 4‐point scale ranging from 1 = *Totally agree* to 4 = *Totally disagree*. Both items reference the same two risk domains (i.e., health and environment) that were also used to measure risk‐benefit perceptions of chemicals in extant research (Saleh et al., 2019). Both items correlated highly (*r* = 0.69) and were averaged to form an index of ARC (Cronbach's alpha = 0.82). Scores were reverse coded so that higher values indicate a higher ARC (see Table 2 for descriptive information).

**TABLE 2:** Descriptive statistics.

Variable	*N*	Mean or %	SD	Min.—Max. or categories
*Individual level*				
ARC	27,042	3.33	0.69	1–4
Age (in years)	27,042	52	18	15–98
Female	27,042	54%		1 = yes; 0 = no
Education	26,611	14%		up to 15 years
		44%		16–19 years
		36%		20 years or more
		6%		Still studying
Problems paying bills	26,690	68%		Almost never/never
		24%		From time to time
		8%		Most of the time
*Country level*				
UA (GLOBE)	17	4.2	0.6	3.2–5.1
UA (Hofstede)	27	71	22	23–100
GDP	28	48,726	19,964	25,312–124,591
GDP (w/o outliers)^a^	26	44,233	10,589	25,312–62,090
Individualism	27	59	18	27–89
Chemical waste	28	68	200	4–1083
Chemical waste (w/o outlier)^b^	27	31	22	4–84
Chemical industry	25	1.4	0.9	0.1–3.2
Trust in cons. protection	28	64.6	13.3	33.4–86

*Note*: All descriptive statistics are based on untransformed variables. Only participants for which data on ARC is available were included. ARC = affective reactions to chemicals; UA (GLOBE) = GLOBE's index of uncertainty avoidance; UA (Hofstede) = Hofstede's index of uncertainty avoidance; Chemical industry = importance of the chemical industry in % of total output of a country; Trust in cons. protection = trust in public authorities to protect consumer rights.

^a^ Ireland (GDP = 89,684) and Luxemburg (GDP = 124,591) are outliers.

^b^ Estonia (1083 kg/capita) is an outlier.

Worry is a typical affective reaction toward hazards (Loewenstein et al., 2001). Measures of worry about a hazard have been used in extant research on risk perception (Siegrist & Árvai, 2020) as well as in research on chemicals (Jansen et al., 2020; Rembischevski et al., 2022).

#### 2.2.2 Uncertainty avoidance

Hofstede's UA scores were downloaded from his website (Hofstede, 2015). Scores range from 0 to 100 where higher scores indicate higher UA in a given country. GLOBE's UA values scores were adopted from their website (GLOBE, 2004a). GLOBE measures UA on a scale ranging from 1 to 7 where higher scores indicate stronger UA values in a given society (House et al., 2004). Similarities and differences between Hofstede and GLOBE in terms of the conceptualization and measurement of UA are discussed in Sections 1.2 and 1.3. Information on Hofstede's and GLOBE's methodology (e.g., measurement instruments, samples used, etc.) can be found in their books on their cultural value dimensions (Hofstede, 2001; House et al., 2004). Hofstede's scores were divided by 100 and GLOBE's by 10 to facilitate the interpretation of coefficients.

#### 2.2.3 Individual‐level control variables

Participants’ age was measured in years. Age was divided by 100. Participants self‐reported their gender as either *man* or *woman*. This variable was dummy coded as 1 = female and 0 = male. Participants’ education was captured by asking them “How old were you when you stopped full‐time education?”. Adopting the Eurostat coding scheme, we coded this variable into the following categories: 1 = up to 15 years; 2 = 16–19 years; 3 = 20 years or older; 4 = still studying. The first category also included participants who have no full‐time education. Category 2 (i.e., 16–19 years) was used as reference category. Participants financial situation was measured using the following question: “During the last twelve months, would you say you had difficulties to pay your bills at the end of the month…?”. Answer options were 1 = *Most of the time*, 2 = *From time to time*, and 3 = *Almost never∖never*. “*Almost never∖never*” was used as reference category.

#### 2.2.4 Country‐level control variables

We used the GDP per capita in PPP for the year 2019 as an indicator of national affluence. GDP data was downloaded from the World Bank website (The World Bank, 2021). We log‐transformed the GDP and divided the resulting log GDP by 10 before it was used in the statistical models.

Individualism scores were taken from Hofestede's website (Hofstede, 2015). Individualism scores range from 0 to 100 where higher scores indicate a stronger individualism and lower scores a stronger collectivism orientation of a society (Hofstede et al., 2010). Scores were divided by 100.

The amount of chemical waste produced in a country was measured in kilograms per capita. Because data for 2019 was not available since this data is collected only biannually, we used data for 2018. This statistic was downloaded from the Eurostat website (Eurostat, 2022a). The importance of the chemical industry in a country was measured as the percentage of the total output of a country that can be attributed to the chemicals and chemical products industry. Information on the importance of the chemical industry for the year 2019 was again downloaded from the Eurostat website (Eurostat, 2022b). Statistics on chemical waste were divided by 100 and on importance of the chemical industry by 10.

Lastly, we adopted data on consumers’ trust in consumer protection from a report by the European Commission: Consumers, Health, Agriculture and Food Executive Agency (2019). This report formed the basis for the EU's 2019 Consumer Conditions Scoreboard (European Commission: Directorate‐General for Justice & Consumers, 2019). This indicator represents the estimated % of consumers in a country who trust public authorities to protect their rights. Because data on this indicator is collected biannually, we used data for 2018 in our analyses. Scores were divided by 100 before they were entered into our statistical models. Table 2 contains descriptive statistics for all measures and Table 3 correlations among country‐level variables.

**TABLE 3:** Zero‐order correlations among the country‐level variables.

	1	2	3	4	5	6
1. UA (GLOBE)						
2. UA (Hofstede)	.65^**^					
3. log GDP^a^	−.84^***^	−.46^*^				
4. Individualism	−.50^*^	−.56^**^	.60^**^			
5. Chemical waste^a^	−.33	−.12	.50^*^	.39^*^		
6. Chemical industry	−.72**	−.12	.50^*^	.39	.57^**^	
7. Trust in cons. protection	−.56^*^	−.44^*^	.61^**^	.58^**^	.34	.13

*Note*: UA (GLOBE) = GLOBE's index of uncertainty avoidance; UA (Hofstede) = Hofstede's index of uncertainty avoidance; Chemical industry = importance of chemical industry; Trust in cons. protection = trust in public authorities to protect consumer rights.

^a^ Outliers excluded (see Table 2 for details).

* *p* < 0.05.

** *p* < 0.01.

*** *p* < 0.001.

### 2.3 Empirical strategy

The effect of UA on consumers’ ARC was tested using multilevel models. This type of model allowed us to model data where consumers *i* are nested in countries *j* and to test the effects of variables on the country level on consumers’ ARC (Nezlek, 2008). Our model took the following form:

(1) $$\text{Level}\;1:\;\mathrm{AR}C_{\mathrm{ij}}=\beta X_{\mathrm{ij}}+\nu_{j}+e_{\mathrm{ij}}$$

(2) $$\text{Level}\;2:\;{\hat{\nu }}_{j}=\alpha +\gamma Z_{j}+u_{j}$$

We used a two‐stage approach where between‐country differences in ARC are estimated in a first stage (Equation 1). These estimated between‐country differences are then used as the dependent variable in the second‐stage models (i.e., Equation 2). The second‐stage models thereby test our predictions on how between‐country differences can be explained. Equation (1) describes a fixed effects model where $\nu_{j}$ is a country fixed effect that captures differences in consumers’ ARC between countries. This model also includes a vector of control variables *X_ij_*, their coefficients *β*, and an error term *e_ij_*. Based on Equation (1), we estimated the ARC country fixed effect $\hat{\nu }$ *_j_* (i.e., estimated marginal means) for the full set of countries. Equation (2) includes these estimated between‐country differences in ARC (i.e., $\hat{\nu }$ *_j_*) as dependent variable, a vector of country level variables *Z_j_* and their coefficients γ, an intercept term *α*, and an error term *u_j_* (Angrist & Pischke, 2009; Bryan & Jenkins, 2016).

Modeling approaches similar to the one used in this research are common in research that analyzes clustered survey data (see e.g., Angrist & Pischke, 2009; Martin, 2023; Twenge et al., 2018). Additional details on our empirical strategy are provided in Section A and information on the software used in Section B in the Supplementary Materials.

In addition, we estimated the UA effect (i.e., second‐stage equation) using instrumental variable regression. This technique identifies variance in UA that is not related to the model's error term. This exogenous portion of the variance in UA is then used to test the UA—ARC relationship. Because of this, instrumental variable estimates are unbiased even if omitted variables or other sources of endogeneity were a concern (Angrist & Pischke, 2009; Antonakis et al., 2010). We discuss our instrumental variable models in detail in Section D in the Supplementary Materials.

## 3 RESULTS

### 3.1 Between‐country variation in affective reactions to chemicals

In line with extant research, consumers in all countries experienced negative ARC on average. That is, consumers in all countries scored above the ARC scale midpoint of 2.5 (*p* < .001) on average. However, there was noticeable variation in ARC between countries. Scores ranged from 3.07 in Finland to 3.6 in Greece. Cyprus was an outlier with a score of 3.81. When controlling for individual‐level variables (see Table C.1 in the Supplementary Materials), country scores ranged from 2.97 in Finland to 3.61 in Greece, with Cyprus again being an outlier (score: 3.76).

### 3.2 Uncertainty avoidance and affective reactions to chemicals

Next, we tested whether UA explains between‐country variation in ARC. The UA effects were statistically significant in the predicted direction. Consumers in higher UA societies seem to have more negative ARC than consumers in lower UA societies (Table 4). This supports our theorizing.

**TABLE 4:** Second‐stage models of affective reactions to chemicals.

	Model 1	Model 2	Model 3^a^	Model 4	Model 5	Model 6^a^	Model 7
Intercept	2.609^***^	2.379^***^	2.523^***^	3.155^***^	3.018^***^	3.008^***^	2.412^***^
	(0.190)	(0.227)	(0.202)	(0.079)	(0.092)	(0.091)	(0.217)
UA (GLOBE)	1.704^**^	2.094^**^	1.716^**^				1.905^**^
	(0.475)	(0.574)	(0.516)				(0.580)
UA (Hofstede)				0.251^*^	0.349^*^	0.339^*^	0.067
				(0.110)	(0.129)	(0.124)	(0.116)
First stage contr.	No	Yes	Yes	No	Yes	Yes	Yes
Countries	17	17	16	27	27	25	17
*R* ^2^	0.503	0.538	0.439	0.179	0.257	0.256	0.544

*Note*: Unstandardized regression coefficients are reported with robust standard errors in brackets; UA (GLOBE) = GLOBE's index of uncertainty avoidance; UA (Hofstede) = Hofstede's index of uncertainty avoidance; First stage contr. = first‐stage model was estimated with (i.e., yes) or without (i.e., no) control variables.

^a^ Influential data points excluded based on Cook's Distance with a cut‐off value of 4/n (Schmidt‐Catran et al., 2019).

* *p* < 0.05.

** *p* < 0.01.

*** *p* < 0.001.

**FIGURE 1:**
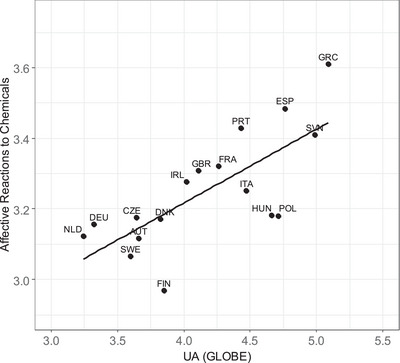
Association between uncertainty avoidance and affective reactions to chemicals. *Note*: See Table 1 for country codes; Affective reactions to chemicals = estimated marginal means based on first‐stage fixed effects model with controls (see Equation 1); UA (GLOBE) = GLOBE's uncertainty avoidance index; Line is best fitting regression line (see Equation 2).

Since outliers may be a concern, we tested whether the UA effects are stable. This was the case. We identified influential observations using Cook's Distance with a cut‐off value of 4/n (Schmidt‐Catran et al., 2019). The UA effects remained statistically significant when influential data points were excluded from the analysis (Models 3 and 6). GLOBE's UA measure thereby predicted more variation in ARC than Hofstede's. When both measures were included in one model, only GLOBE's measure remained statistically significant (Model 7). As expected, this suggests that the rule orientation component of UA drives UA—ARC effects. The anxiety component of UA, which is prominently featured in Hofstede's but not included in GLOBE's UA index, does not seem to explain variation in ARC. Taken together, GLOBE's UA index is theoretically (see Section 1.3.1) and practically more relevant for predicting ARC than Hofstede's UA index. We will, therefore, focus on GLOBE's UA index in the remaining analyses. The relationship between GLOBE's UA index and ARC (Model 2) is visualized in Figure 1.

We also found support for alternative explanations of between‐country variation in ARC (Table 5). Consumers reported less negative ARC in more affluent and individualistic societies, and in societies where chemicals were more prevalent (i.e., more chemical waste per capita and greater importance of the chemical industry) and where trust in consumer protection was stronger.^3^

**TABLE 5:** Second‐stage models of affective reactions to chemicals (first stage with controls).

	Model 1	Model 2^a^	Model 3	Model 4	Model 5^a^	Model 6	Model 7
Intercept	4.892^***^	5.774^***^	3.517^***^	3.301^***^	3.402^***^	3.469^***^	3.680^***^
	(1.267)	(1.206)	(0.080)	(0.142)	(0.044)	(0.072)	(0.148)
GDP (log)	−1.500	−2.349^*^					
	(1.185)	(1.130)					
Individualism			−0.432^**^				
			(0.131)				
Chemical waste				−0.029	−0.410^**^		
				(0.456)	(0.115)		
Ch. industry						−1.243^**^	
						(0.371)	
Trust							−0.616^*^
							(0.226)
Countries	28	26	27	28	26	25	28
*R* ^2^	0.082	0.187	0.259	0.108	0.368	0.345	0.218

*Note*: Unstandardized regression coefficients are reported with robust standard errors in brackets; GDP (log) = log‐transformed GDP; Ch. industry = importance of chemical industry; Trust = trust in consumer protection.

^a^ Influential data points excluded based on Cook's Distance with a cut‐off value of 4/n (Schmidt‐Catran et al., 2019).

* *p* < 0.05.

** *p* < 0.01.

*** *p* < 0.001.

Next, we tested whether UA explained ARC beyond the alternative explanations. UA remained statistically significant in all models with one exception (Table 6). UA was no longer statistically significant in the model that included the importance of the chemical industry as a control variable (Model 5). However, the effect of the importance of the chemical industry was not statistically significant either in that model and the number of countries was extremely small (*N* = 12) which might have made it difficult to detect effects. Overall, these results suggest that UA is a unique predictor of between‐country variation in ARC and that its effect is not due to variation in other societal characteristics.

**TABLE 6:** Second‐stage models of affective reactions to chemicals (first stage with controls).

	Model 1	Model 2	Model 3	Model 4	Model 5^a^	Model 6
Intercept	1.476	2.687^***^	2.663^***^	2.587^***^	2.692^***^	3.003^***^
	(1.422)	(0.317)	(0.155)	(0.461)	(0.553)	(0.270)
UA (GLOBE)	2.331^***^	1.710^*^	1.712^**^	1.763^*^	1.554^b^	1.464^*^
	(0.546)	(0.611)	(0.427)	(0.788)	(0.925)	(0.507)
GDP (log)	0.745					
	(1.239)					
Individualism		−0.240				
		(0.188)				
Chemical waste			−0.339^**^			
			(0.106)			
Ch. industry				−0.419	−0.645	
				(1.184)	(1.442)	
Trust						−0.534^*^
						(0.217)
Countries	17	17	17	14	12	17
*R* ^2^	0.545	0.592	0.687	0.534	0.424	0.647

*Note*: Unstandardized regression coefficients are reported with robust standard errors in brackets; UA (GLOBE) = GLOBE's index of uncertainty avoidance; GDP (log) = log‐transformed GDP; Ch. industry = importance of chemical industry; Trust = trust in consumer protection.

^a^ Influential data points excluded based on Cook's Distance with a cut‐off value of 4/n (Schmidt‐Catran et al., 2019).

^b^ *p* = 0.13.

* *p* < 0.05.

** *p* < 0.01.

*** *p* < 0.001.

Because it is possible that there are alternative explanations for the UA effect that we have not identified, we estimated the UA effect using instrumental variable regression (Table 7). The UA effect is statistically significant in all instrumental variable models. Moreover, Wu−Hausman tests are not statistically significant. This indicates that endogeneity (e.g., due to omitted variables) is not a concern and that the original estimates (see Table 4) can be trusted (see Section D in the Supplementary Materials for a detailed discussion).

**TABLE 7:** Second‐stage instrumental variable models of affective reactions to chemicals (first stage with controls).

	Model 1		Model 2		Model 3	
	A	B	A	B	A	B
	
DV	ARC	UA	ARC	UA	ARC	UA
Intercept	2.464^***^	0.551^*^	2.376^***^	0.155	2.441^***^	0.475^***^
	(0.250)	(0.226)	(0.252)	(0.098)	(0.264)	(0.113)
$\hat{\mathrm{UA}}$ ^a^	1.888^**^		2.100^**^		1.943^*^	
	(0.632)		(0.632)		(0.662)	
*IVs*						
FTO		−0.752^**^				−0.570^**^
		(0.239)				(0.182)
PDI		0.309		−0.020		
		(0.293)		(0.305)		
COL				0.590^*^		0.349^*^
				(0.200)		(0.123)
Equation	D.1a	D.1b	D.1a	D.1b	D.1a	D.1b
Countries	17	17	17	17	17	17
*R* ^2^	0.533	0.732	0.538	0.650	0.535	0.797
Strong IVs	*F*(2, 14) = 17.5; *p* < 0.001		*F*(2, 14) = 11.7; *p* = 0.001		*F*(2, 14) = 23.7; *p* < 0.001	
Wu−Hausman test	*F*(1, 14) = 0.45; *p* = 0.52		*F*(1, 14) = 0.00; *p* = 0.98		*F*(1, 14) = 0.29; *p* = 0.60	
Sargan test	*Chi^2^* (1) = 0.28; *p* = 0.60		*Chi^2^* (1) = 0.01; *p* = 0.91		*Chi^2^* (1) = 0.40; *p* = 0.53	

*Note*: Unstandardized regression coefficients are reported with robust standard errors in brackets. DV = dependent variable; ARC = affective reactions to chemicals; UA = GLOBE's index of uncertainty avoidance; IVs = instrumental variables; FTO = future orientation practices; PDI = power distance practices; COL = ingroup collectivism practices.

^a^ Predicted using Equation (D.1b) (see Models 1B, 2B, and 3B for the coefficients of the IVs).

* *p* < 0.05.

** *p* < 0.01.

*** *p* < 0.001.

## 4 DISCUSSION

We studied between‐country variation in ARC (i.e., to chemicals in general not focusing on any specific chemical substance) in the present research. Previous research has indicated that ARC (i.e., chemophobia) may vary between countries (Bearth et al., 2019). We build on and extend this research in several important ways. First, we included more countries in our analysis (i.e., 28 compared to eight) and we studied how this between‐country variation can be explained. The latter contributes to a more comprehensive understanding of how consumers form ARC and particularly how this is influenced by different features of the societal context in which consumers are embedded in.

In our sample, all countries scored above the scale midpoint which indicates that consumers in all countries in our dataset have negative ARC on average. This is in line with extant research that indicates that consumers typically hold negative views of chemicals in general (Siegrist & Bearth, 2019). Replicating Bearth and colleagues’ (2019) result, we found practically significant variation in ARC between countries. The countries with the most negative ARC on average scored about 0.6 higher on a scale ranging from 1 to 4 than the countries with the least negative ARC. These differences remained almost unchanged when we controlled for individual‐level demographic variables. This indicates that between‐country differences in ARC are due to differences in the societal context rather than due to differences in the composition of consumer populations.

In line with our theorizing, our results suggest that this variation can be explained, at least in part, by between‐country differences in UA. As predicted, consumers reported more negative ARC in higher UA societies compared to lower UA societies. This finding is in line with extant research which found that higher UA was associated with stronger negative affective reactions to hazards and threats in other domains (Lu, 2023; Rojas‐Méndez, 2021). This finding also suggests that between‐country variation in ARC is at least to some extent systematic (i.e., varies in a meaningful way) rather than random as it can be explained by cultural differences based on extant theory.

GLOBE's UA values scores thereby explained substantially more variation in ARC than Hofstede's UA index. When both measures were entered in a model simultaneously, only GLOBE's index remained statistically significant. Considering the differences between both UA measures (see Section 1.3.1 for a detailed discussion), this indicates that variation in ARC scores is not driven by societal differences in anxiety. Rather, between‐country differences in ARC seem to be due to how much societies wish to avoid failures and losses, which is captured by the rule orientation component of UA. The latter is only one of the components in Hofstede's index, while it is the sole component in GLOBE's measure (Venaik & Brewer, 2010). This is in line with extant theorizing that suggests that consumers in higher UA societies may value predictability to avoid potential losses and may in general focus more on potential losses than consumers in lower UA societies because of this (Bontempo et al., 1997). This seems to translate into differences in ARC. Our findings suggest that consumers in higher UA societies worry more about the potential dangers of chemicals than consumers in lower UA societies. In other words, UA seems to magnify negative ARC.

Our results also indicate that the UA—ARC relationship cannot be explained by other features of societies. The UA effects remained statistically significant in all but one model when controls were included. However, in that one model (i.e., the model that controlled for the importance of the chemical industry), none of the coefficients was statistically significant. A likely explanation for this is that the number of countries (i.e., 12) was too small to detect effects. Importantly, the UA effect was statistically significant in our instrumental variable models. This indicates that endogeneity (e.g., due to omitted variables) is not a concern and that the UA effect is not spurious (i.e., explained by variation in other features of societies).

Even if the effects of some of our control variables were no longer statistically significant when UA was included in a model, these effects may still be of practical and theoretical relevance (Martin, 2023; Tam & Chan, 2018). In our dataset, as is often the case in multicountry studies, the sample size was small (i.e., between 17 and 12 countries depending on the variables included), while many of the predictors were highly correlated (see Table 3). This made it difficult to reliably separate the effects of individual predictors (Tam & Chan, 2018). It is, therefore, permissible to focus on results based on single‐predictor models (Aguinis et al., 2013).

While the emphasis of this research was on UA, the control variables also explained variation in ARC. We will discuss their effects in the following focusing on the results of our single‐predictor models. First, ARC were less negative in individualistic and affluent countries. This is in line with research that suggests that these societies may view innovations more positively than collectivistic and less affluent societies (Gorodnichenko & Roland, 2011). It is possible that more positive attitudes toward innovations and technologies reduce consumers’ concerns about chemicals to some extent.

We also found that ARC were less negative in countries where chemicals were more prevalent (i.e., measured as the importance of the chemical industry and amount of chemical waste per person). This is in line with research on the typhoon eye effect that suggests that consumers may be less concerned about hazards if these hazards are spatially more proximal. Such effects have been found for nuclear energy, for example (Latré et al., 2017; Pampel, 2011). It has been argued that this is because consumers in these countries are more familiar with this technology and are, therefore, less worried about it (Pampel, 2011). The same may be the case for chemicals. Consumers may be more familiar with chemicals in countries where there is more chemical waste and where the chemical industry is more important. Such an explanation would be in line with research that suggests that knowledge of toxicological principles reduces fear of chemicals (Bearth et al., 2019; Saleh et al., 2019).

Lastly, we also found that trust in consumer protection was associated with less negative ARC. This is in line with extant findings on the individual level that suggest that consumers who trust regulators more experience less fear of chemicals (Bearth et al., 2019; Saleh et al., 2019). Our results go beyond these extant findings on the individual level, however. The level of trust was not reported by the participants in our dataset but was adopted from a different dataset. This suggests that trust in consumer protection also operates on the societal level and that members of a society hold a shared trust in consumer protection. This may be similar to how cultural values form and are socialized to members of a society. For example, older generations may pass on knowledge and experiences to younger generations. This wisdom in addition to individual experiences then forms the basis for shared beliefs which are then again transmitted to the next generation of consumers in a society (Inglehart, 2007; Kesebir et al., 2010).

### 4.1 Implications

Because negative ARC may have undesirable consequences, such as consumers avoiding beneficial products, it has been suggested to use educational interventions to enable consumers to make better decisions based on facts rather than on gut feelings (Bearth et al., 2019). It appears to be difficult to design such interventions, however (Hartings & Fahy, 2011). Our research has implications for how these interventions can be fine‐tuned to potentially be more effective in different contexts. First, research suggests that consumers in higher UA societies may focus primarily on risks while discounting benefits when evaluating hazards (Bontempo et al., 1997). Accordingly, if one wishes to counteract negative ARC in contexts where this would benefit consumers, particularly in higher UA societies, information‐based interventions should focus on educating consumers about risks rather than benefits. This is because consumers may not be receptive to information on benefits as benefits may be of little relevance in their overall evaluation of chemicals in higher UA societies.

Second, consumers have been found to focus on the severity of threats rather than on their likelihood of materializing when they have strong emotional reactions to a hazard (Sunstein, 2003). This indicates that information‐based interventions with the goal of nurturing fact‐based risk perceptions (i.e., risk as analysis vs. risk as feelings) should educate consumers about the magnitude of typical risks rather than about how unlikely worst‐case scenarios are. Consumers had negative ARC on average in all countries in the dataset. This recommendation, therefore, appears to be relevant in many different countries. However, this strategy may be particularly effective in higher UA societies where ARC appear to be most negative.

### 4.2 Limitations and future research

The present research contributes to a better understanding of ARC and particularly of how UA and other societal characteristics are associated with more or less negative ARC. However, this research shares the typical limitations of multinational comparative research (Fairbrother et al., 2019; Hornsey et al., 2019; Martin, 2023).

First, the dataset is based on a nonrandom sample of countries. Due to this, it is not clear whether the findings generalize to other sets of countries (Schmidt‐Catran et al., 2019). Given that our findings are in line with extant research that has been supported in other samples of countries, we believe that our findings are generalizable. Moreover, given the similarity between the countries due to their geographic proximity and membership in the European Union, we would expect to find stronger UA effects in more diverse sets of countries with more variation in UA.

Second, it is not clear whether participants in different countries interpreted the ARC measure in the same way (Davidov et al., 2014; Hornsey et al., 2019). However, our results are in line with similar research on concerns about the COVID pandemic (Rojas‐Méndez, 2021) and vaccines (Lu, 2023). In addition, the findings appear to be systematic (i.e., between‐country differences in ARC are meaningfully associated with variation in other country‐level variables). This suggests that it is unlikely that the between‐country differences are a methodological artifact. Moreover, it has been suggested to model between‐country differences using substantive predictors of these differences in cases where measurement equivalence is a concern (Boer et al., 2018; Davidov et al., 2012). This is exactly what we did. We, therefore, believe that our approach to modeling the data constitutes a meaningful test of our theoretical predictions.

Our measure of ARC referenced “chemicals present in everyday products.” Our findings are, therefore, limited to consumers’ ARC to chemicals in general. Since ARC has been found to be related to the risk perception of specific chemicals and products (Buchmüller et al., 2020; Saleh et al., 2021), we would expect that UA also explains between‐country variation in the perception of specific chemicals. However, future research is needed to test the UA—ARC relationship in the context of different types of chemicals.

Lastly, it is not clear whether between‐country differences may change over time. Our research indicates that between‐country variation in ARC is largely due to cultural differences in UA. Cultural differences tend to be stable (Beugelsdijk et al., 2015). We would, therefore, expect that between‐country differences in ARC will also be stable over time. This, however, is speculative. Future research could test this using longitudinal designs.

## 5 CONCLUSIONS

Our study contributes to a better understanding of affective reactions to chemicals in general. In particular, our results constitute first insights into how UA and other characteristics of the societal context contribute to shaping consumers’ ARC. In addition to its theoretical contribution, our research also has implications for the design of interventions that can be used to nurture fact‐based risk‐benefit perceptions of chemicals in consumers and help to reduce adverse consequences of negative ARC. However, much more is to be learned about ARC and consumers’ risk perceptions of chemicals more widely and future research in these areas is encouraged. For example, future research may be able to identify additional societal characteristics that influence ARC or could study contextual variables at a group or community level.

## CONFLICT OF INTEREST STATEMENT

The author declares no conflicts of interest.

## Supporting information



Supporting Information

## Data Availability

The data and materials are freely available on the internet (see Methods section for details).

## Footnotes

1 Some authors refer to this component as “anxiety” (Minkov & Hofstede, 2014) and others as “stress” (Venaik & Brewer, 2010). We will refer to it as “anxiety.”

2 Our discussion here refers to the GLOBE cultural UA values rather than practices. The values and practices scores correlate negatively (Javidan et al., 2006). Different explanations have been offered for the apparent differences between practice and value scores (Javidan et al., 2006; McCrae et al., 2008). We used the values scores in this research. While extant theory explains why values may influence consumers’ ARC (see Section 1.2), it is not clear why cultural practices (i.e., how consumers actually behave) would explain differences in affective reactions and judgments.

3 For all models in Tables 5−7, we checked for influential data points using Cook's Distance with a cut‐off value of 4/n (Schmidt‐Catran et al., 2019). For the sake of parsimony, we report only those models with outliers excluded where the interpretation of results is substantively different from the model with all observations included (i.e., at least one statistically significant effect is no longer statistically significant or vice versa). Where there was a difference, we focus on the models with outliers excluded in our discussion of the results.
