# Supplementary material for: Cultural uncertainty avoidance predicts consumers’ affective reactions to chemicals
Source: Risk Anal. 2025 Jan 17;45(7):1792–805. doi: 10.1111/risa.17693 (PMC12396941; doi:10.1111/risa.17693)
Supplement: Supplementary file 1 — Supporting Information [file RISA-45-1792-s001.pdf]

Cultural Uncertainty Avoidance Predicts Consumers' Affective Reactions to Chemicals

Supplement Material

A: ADDITIONAL DETAILS ON AND RATIONAL FOR EMPIRICAL STRATEGY

The first stage model (Equation 1) was estimated using ordinary least squares (OLS). Country differences in ARC were modelled using dummy variables for the different countries. Because the effects of individual level controls in Equation 1 may vary between countries, we used cluster robust standard errors (Cameron & Miller, 2015; Heisig & Schaeffer, 2019).

In our second stage models (Equation 2), we modelled the effects of UA without controls first (i.e., single-predictor models) and then with one control variable at a time (i.e., multi-predictor models). We did not run models with more than two variables. This is because it may be difficult to statistically separate the effects of two or more correlated variables when the number of countries is small (Tam & Chan, 2018). It has therefore been deemed acceptable to use single-predictor models for testing theory in such cases (Aguinis, Gottfredson, & Culpepper, 2013). Our approach to using control variables is in line with best practice recommendations. The literature on control variables recommends to theoretically justify why given control variables may be relevant and to then treat and test them as alternative hypotheses one-by-one (Becker, Atinc, Breaugh, & Carlson, 2016; Spector, 2021; Spector & Brannick, 2011). For example, Spector (2021, p. 743) recommends that "once the baseline X-Y relationship is established, add controls one-by-one to rule them in or out as

potential explanations". The theoretical rational for why each control variable was included is discussed in Section 1.3.2. in the manuscript.

Different methods for estimating the second stage model (Equation 2) have been suggested and used in the literature, such as Feasible Generalized Least Squares (FGLS) and OLS (Angrist & Pischke, 2009; Lewis & Linzer, 2005). We used OLS because it has been recommended for analyses where the number of countries is small (Bryan & Jenkins, 2016), which is the case in our dataset. The dependent variable in Equation 2 is an estimated dependent variable. This introduces heteroscedasticity (Donald & Lang, 2007). To address this, we used robust standard errors (Lewis & Linzer, 2005).

As mentioned, our models were estimated using a two-stage process (i.e., Equation 1 was estimated first, and the estimated fixed effect was then used in Equation 2 in a second stage). We could have estimated both equations simultaneously using random effects (RE) models. We have chosen to use two-stage models instead of RE models because two-stage models have a number of desirable properties. In particular, we favor two-stage models over RE models because they tend to be more stable (i.e., less likely to produce biased or unstable estimates), especially when the number of higher-level units is small, and can be visualized easily (Bryan & Jenkins, 2016; Wooldridge, 2003). In addition, the two-stage approach makes the relevant sample size and its restrictions more obvious. That is, they make obvious that the relevant sample size when testing country level effects is the number of countries, not the number of participants and that therefore the number of variables that can be included in the higher-level equation (i.e., country level equation in our case) is limited by the number of countries. While this is the case for RE and two-stage models, it is more obvious when using two-stage models (Bryan & Jenkins, 2016; Snijders, 2005). Two-stage models also do not make the random effects assumption (i.e., a FE model is used in the first stage), which means that individual level coefficients are interpretable, while this is not necessarily the case for

RE models (Antonakis, Bastardoz, & Rönkkö, 2021; Bell, Fairbrother, & Jones, 2019; Enders & Tofighi, 2007).

The main drawback of this approach, compared to a random effects (RE) model, is that in a scenario where the assumptions of RE models are met and where the number of units within groups is small (i.e., only few consumers were sampled per country) and the number of groups (i.e., countries) is large, RE models can be more efficient (i.e., smaller SEs) than two-stage models (Heisig, Schaeffer, & Giesecke, 2017; Oshchepkov & Shirokanova, 2022). However, particularly in contexts where the number of higher level units is small (i.e., number of countries in our case), RE models may produce anti-conservative findings (Bryan & Jenkins, 2016).

A recent literature review compared RE models, which they refer to as multilevel models (MLM) and hierarchical models with the two-stage method, which we use and which they refer to as 'economic solution' or estimated dependent variable (EDV) models (Oshchepkov & Shirokanova, 2022). They conclude that "in practice, MLM's higher efficiency is contingent on the research goal and the data used. In cases when the number of observations within groups is small relative to the number of groups, MLM produces more efficient estimates as it uses both within- and between-group information. In cases where considerable information is available within the groups relative to the number of groups, 'the added effort of fitting a more complex single-stage linear hierarchical model would provide little advantage relative to the simple two-stage EDV method'(Lewis & Linzer, 2005)" (Oshchepkov & Shirokanova, 2022, pp. 12 & 13). Overall, given the desirable properties of two-stage models we favor two-stage models over RE models in the context of this research.

B: SOFTWARE USED

Analyses were performed in R (R Core Team, 2021) using the sandwich (Zeileis, 2004, 2006), lmtest (Zeileis & Hothorn, 2002), estimatr (Blair, Cooper, Coppock, Humphreys, & Sonnet, 2022), emmeans (Lenth, 2022), and the ivreg (Fox, Kleiber, & Zeileis, 2024) packages in addition to R's base and stats packages. The car package (Fox & Weisberg, 2019) was used for recoding variables. The Hmisc (Harrell Jr, 2021) and psych (Revelle, 2021) packages were used for correlation analyses and descriptive statistics respectively. Regression tables were created using the stargazer package (Hlavac, 2018) and correlation tables with the scipub package (Pagliaccio, 2021). The ggplot2 (Wickham, 2016) and ggrepel packages (Slowikowski, 2020) were used to create the figure.

C: ADDITIONAL STATISTICAL RESULTS

Table C.1. First Stage Fixed Effects Model of Affective Reactions to Chemicals

**Independent variables**	**Coef. (SE)**
Age	0.048 (0.039)
Female	0.136*** (0.014)
Education	
up to 15 years	-0.073** (0.019)
16 – 19 years (ref.)	
20 years or more	0.044** (0.013)
Still studying	-0.039 (0.034)
Problems paying bills	
Almost never/never (ref.)	
From time to time	-0.103*** (0.022)
Most of the time	-0.138** (0.042)
Country FE	Yes
Observations	26,276
R^2	0.061

Note. *) p < 0.05; **) p < 0.01; ***) p < 0.001; Unstandardized regression coefficients are reported with clustered standard errors in brackets; Country FE = country fixed effect.

D: INSTRUMENTAL VARIABLE ANALYSIS

Description and Econometric Details

Instrumental variable regression uses instrumental variables to identify variation in the focal predictor (i.e., UA) that is unrelated to a model's error term. Only this portion of the variance is then used to estimate the effect of UA, rather than the full variance of UA. Because only exogenous variance in UA is used, the estimated coefficients are consistent. This means that coefficients are unbiased even if endogeneity due to omitted variables, simultaneity, etc. is a concern (Angrist & Pischke, 2009; Antonakis, Bendahan, Jacquart, & Lalive, 2010).

Equations D.1a and D.1b describe our second stage (i.e., country level) instrumental variable model.

Level 2: $\hat{v}_j = \alpha_{IV} + \gamma_{IV}\widehat{UA}_j + u_{IVj}$ (D.1a)

Level 2: $UA_j = \pi + \lambda Q_j + r_j$ (D.1b)

Equation D.1a is similar to Equation 2 as described in the manuscript. The main difference is that not the full information on UA was used but only the exogeneous portion of its variance (i.e., $\widehat{UA}_j$). The latter was estimated using Equation D.1b. Equation D.1b includes an intercept term π, a vector of instrumental variables Q, their coefficients λ, and an error term r. Both equations were estimated simultaneously using the "ivreg" command in R using the "2SLS" estimator (Fox et al., 2024). This estimator is the most commonly used instrumental variable estimator (Antonakis et al., 2010).

Instrumental variable regression produces consistent and unbiased coefficients only if valid instrumental variables are used. Instrumental variables, thereby, need to be correlated with UA and cannot be correlated with ARC other than through their relationship with UA (Antonakis et al., 2010; Bollen, 2012). Both requirements can be tested. First, we tested whether the instruments predicted UA well using an F-test. Whether our instrumental variables are uncorrelated with ARC except due to their correlation with UA (i.e., the exclusion restriction) can be tested using a Sargan test. This test can be performed only when a model includes more instrumental variables than (potentially) endogenous predictors (Antonakis et al., 2010; Bollen, 2012). Because of this, despite the small sample size, we used two instrumental variables per model to instrument UA. Because we needed only two instrumental variables to perform Sargan tests, while we have identified three instrumental variables, we reran our model with different combinations of instrumental variables. The results of both tests are reported in Table 7 in the manuscript. Details of our instrumental variables will be discussed in the following.

Instrumental Variable Selection and Measures

We used auxiliary instrumental variables in our instrumental variable models. These are variables that fulfill the requirements for valid instruments but that are not of interest for any other reason than to instrument UA in the context of our research. In other words, these variables do not play a role in our theoretical model and were included in our statistical models solely for the purpose of instrumenting UA (Bollen, 2012).

Based on House et al.’s (2004) research on cultural values and practices, we have identified three cultural practices that fulfill the requirement of valid instrumental variables (i.e., future orientation practices, power distance practices, and ingroup collectivism

practices). These three cultural practices are strongly related to UA. Consumers in high UA cultures focus on the here and now and avoid planning too far into the future (i.e., low future orientation practices) as the future naturally is less controllable and holds uncertainty. Consumers in high UA societies also tend to form highly structured societies and social groups where every individual has a clearly defined place (i.e., power distance practices), among others, in an attempt to manage and avoid uncertainty. Lastly, consumers in high UA cultures also seek to build strong social groups and networks (i.e., ingroup collectivism practices). This is to secure access to resources and social support among others to prepare themselves for uncertain future events. (House et al., 2004; Weber & Hsee, 1998). We argue that these cultural practices are valid instrumental variables that can be used to identify exogeneous variance in UA, which can then be used to estimate a consistent and unbiased coefficient of the UA effect. First, research suggests that these cultural practices correlate strongly with UA (House et al., 2004). Second, we argue that none of these cultural practices is directly (i.e., other than through UA) related to consumers' ARC. As discussed above, we tested both assumptions (i.e., using *F*-tests and Sargan tests) and present empirical evidence that suggests that both hold (see Table 7 in the manuscript and Discussion section below for more details).

Information on the methodology underlying the three cultural practice indices that we used as instrumental variables can be found in House and colleagues' (2004) seminal book on cultural values and practices. Descriptive statistics and correlations are shown in Tables D.1 and D.2.

Table D.1. Descriptive Statistics of Instrumental Variables

Variable	N	M	SD	Min. – Max.
FTO Practices	17	3.9	0.5	3.1 – 4.6
PDI Practices	17	5	0.6	3.6 – 5.6
COL Practices	17	4.6	0.8	3.2 – 5.5

Note. All descriptive statistics are based on untransformed variables; FTO = Future orientation practices; PDI = Power distance practices; COL = Ingroup collectivism practices.

Table D.2. Zero-Order Correlations of UA and Instrumental Variables

	1	2	3
1. UA (GLOBE)			
2. FTO	-.81***		
3. PDI	.63**	-.49*	
4. COL	.81***	-.64**	.80***

Note. *) $p < 0.05$; **) $p < 0.01$; ***) $p < 0.001$; UA (GLOBE) = GLOBE's index of uncertainty avoidance; FTO = Future orientation practices; PDI = Power distance practices; COL = Ingroup collectivism practices.

Discussion

Our analyses indicate that our instrumental variables predicted UA well. All correlation coefficients exceed 0.62 (Table D.2), the R^2's were larger than 0.64 in all models (Models 1B, 2B, and 3B in Table 7 in the manuscript), and the strong instrumental variable tests were

all statistically significant. It is important to note that, even though the instrumental variables correlate highly with UA in our dataset, research indicates that they are all clearly distinct from UA conceptually and empirically (House et al., 2004).

Even though all instrumental variables correlated strongly with UA (see Table D.2), not all instrumental variables are always statistically significant in all models (Models 1B, 2B, and 3B in Table 7 in the manuscript). However, as discussed with regard to control variables in the manuscript, these regression coefficients need to be interpreted with caution since variables correlated highly and since the sample size was small. Despite this, it was necessary to include at least two instrumental variables in each model in order to perform Sargan tests to test the validity of the instruments.

The Sargan tests suggest that our instrumental variables are valid. That is, there is no evidence that any of our instrumental variables correlated with the error term of the UA – ARC model. In other words, all instrumental variables fulfill the exclusion restriction. If statistically significant, a Sargan test would indicate that at least some instrumental variables would be related to a model's error term and that they would therefore violate the exclusion restriction. However, the p-values of all Sargan tests were well above conventional levels of statistical significance (i.e., $ps > 0.5$; see Table 7).

Lastly, Wu-Hausman tests were computed to test whether the UA coefficient estimated by an instrumental variable regression (Table 7) is different from the coefficient estimated by the regular second stage regression (Model 2 in Table 4). None of our Wu-Hausman tests was statistically significant at conventional levels (i.e., all $p > 0.5$). This indicates that endogeneity (e.g., due to omitted variables) is not an issue and that there is no bias in the UA coefficients. In other words, the coefficients in Table 4 can be trusted.

Overall, the results of the instrumental variable models lend further support to our theoretical prediction that UA explains between-country variation in ARC. Importantly, the results of these models indicate that UA indeed affects ARC and that our estimated UA coefficients are not biased due to omitted variables or endogeneity issues more generally.

E: REFERENCES

Aguinis, H., Gottfredson, R. K., & Culpepper, S. A. (2013). Best-practice recommendations for estimating cross-level interaction effects using multilevel modeling. *Journal of Management*, *39*(6), 1490–1528. https://doi.org/10.1177/0149206313478188

Angrist, J. D., & Pischke, J.-S. (2009). *Mostly Harmless Econometrics: An Empiricist's Companion*. Princeton University Press. https://doi.org/10.2307/j.ctvcm4j72

Antonakis, J., Bastardoz, N., & Rönkkö, M. (2021). On ignoring the random effects assumption in multilevel models: Review, critique, and recommendations. *Organizational Research Methods*, *24*(2), 443–483. https://doi.org/10.1177/1094428119877457

Antonakis, J., Bendahan, S., Jacquart, P., & Lalive, R. (2010). On making causal claims: A review and recommendations. *The Leadership Quarterly*, *21*(6), 1086–1120. https://doi.org/10.1016/j.leaqua.2010.10.010

Becker, T. E., Atinc, G., Breaugh, J. A., & Carlson, K. D. (2016). Statistical control in correlational studies: 10 essential recommendations for organizational researchers. *Journal of Organizational Behavior*, *37*(2), 157–167. https://doi.org/10.1002/job.2053

Bell, A., Fairbrother, M., & Jones, K. (2019). Fixed and random effects models: Making an informed choice. *Quality & Quantity*, *53*(2), 1051–1074. https://doi.org/10.1007/s11135-018-0802-x

Blair, G., Cooper, J., Coppock, A., Humphreys, M., & Sonnet, L. (2022). *estimatr: Fast Estimators for Design-Based Inference.* Retrieved from https://CRAN.R-project.org/package=estimatr

Bollen, K. A. (2012). Instrumental variables in sociology and the social sciences. *Annual Review of Sociology*, *38*, 37–72. https://doi.org/10.1146/annurev-soc-081309-150141

Bryan, M. L., & Jenkins, S. P. (2016). Multilevel modelling of country effects: A cautionary tale. *European Sociological Review*, *32*(1), 3–22. https://doi.org/10.1093/esr/jcv059

Cameron, A. C., & Miller, D. L. (2015). A practitioner's guide to cluster-robust inference. *The Journal of Human Resources*, *50*(2), 317–372. https://doi.org/10.3368/jhr.50.2.317

Donald, S. G., & Lang, K. (2007). Inference with difference-in-differences and other panel data. *Review of Economics and Statistics*, *89*(2), 221–233. https://doi.org/10.1162/rest.89.2.221

Enders, C. K., & Tofighi, D. (2007). Centering predictor variables in cross-sectional multilevel models: A new look at an old issue. *Psychological Methods*, *12*(2), 121–138. https://doi.org/10.1037/1082-989X.12.2.121

Fox, J., Kleiber, C., & Zeileis, A. (2024). *ivreg: Instrumental-Variables Regression by "2SLS", "2SM", or "2SMM", with Diagnostics*. Retrieved from https://CRAN.R-project.org/package=ivreg

Fox, J., & Weisberg, S. (2019). *An {R} Companion to Applied Regression* (3rd ed.). Thousand Oaks, CA: Sage. Retrieved from https://socialsciences.mcmaster.ca/jfox/Books/Companion/

Harrell Jr, F. E. (2021). *Hmisc: Harrell Miscellaneous*. Retrieved from https://CRAN.R-project.org/package=Hmisc

Heisig, J. P., & Schaeffer, M. (2019). Why you should always include a random slope for the lower-level variable involved in a cross-level interaction. *European Sociological Review*, *35*(2), 258–279. https://doi.org/10.1093/esr/jcy053

Heisig, J. P., Schaeffer, M., & Giesecke, J. (2017). The costs of simplicity: Why multilevel models may benefit from accounting for cross-cluster differences in the effects of controls. *American Sociological Review, 82*(4), 796. https://doi.org/10.1177/0003122417717901

Hlavac, M. (2018). *stargazer: Well-Formatted Regression and Summary Statistics Tables*. Retrieved from https://CRAN.R-project.org/package=stargazer

House, R. J., Hanges, P. J., Javidan, M., Dorfman, P. W., & Gupta, V. (2004). *Culture, Leadership, and Organizations: The GLOBE Study of 62 Societies*. Thousand Oaks, CA: Sage.

Lenth, R. V. (2022). *emmeans: Estimated Marginal Means, Aka Least-Squares Means*. Retrieved from https://CRAN.R-project.org/package=emmeans

Lewis, J. B., & Linzer, D. A. (2005). Estimating regression models in which the dependent variable is based on estimates. *Political Analysis*, *13*(4), 345–364. https://doi.org/10.1093/pan/mpi026

Oshchepkov, A., & Shirokanova, A. (2022). Bridging the gap between multilevel modeling and economic methods. *Social Science Research*, *104,* Article 102689. https://doi.org/10.1016/j.ssresearch.2021.102689

Pagliaccio, D. (2021). *scipub: Summarize Data for Scientific Publication*. Retrieved from https://CRAN.R-project.org/package=scipub

R Core Team. (2021). *R: A Language and Environment for Statistical Computing.* Vienna, Austria: R Foundation for Statistical Computing. Retrieved from https://www.R-project.org/

Revelle, W. (2021). *psych: Procedures for Psychological, Psychometric, and Personality Research*. Evanston, Illinois: Northwestern University. Retrieved from https://CRAN.R-project.org/package=psych

Slowikowski, K. (2020). *ggrepel: Automatically Position Non-Overlapping Text Labels with "ggplot2."* Retrieved from https://CRAN.R-project.org/package=ggrepel

Snijders, T. A. B. (2005). Power and Sample Size in Multilevel Linear Models. In B. Everitt & D. Howell (Eds.), *Encyclopedia of Statistics in Behavioral Science* (Vol. 3, pp. 1570–1573). Wiley. https://doi.org/10.1002/0470013192.bsa492

Spector, P. E. (2021). Mastering the use of control variables: The hierarchical iterative control (HIC) approach. *Journal of Business and Psychology*, *36*(5), 737–750. https://doi.org/10.1007/s10869-020-09709-0

Spector, P. E., & Brannick, M. T. (2011). Methodological urban legends: The misuse of statistical control variables. *Organizational Research Methods*, *14*(2), 287–305. https://doi.org/10.1177/1094428110369842

Tam, K.-P., & Chan, H.-W. (2018). Generalized trust narrows the gap between environmental concern and pro-environmental behavior: Multilevel evidence. *Global Environmental Change*, *48*, 182–194. https://doi.org/10.1016/j.gloenvcha.2017.12.001

Weber, E. U., & Hsee, C. (1998). Cross-cultural differences in risk perception, but cross-cultural similarities in attitudes towards perceived risk. *Management Science*, *44*(9), 1205–1217. https://doi.org/10.1287/mnsc.44.9.1205

Wickham, H. (2016). *ggplot2: Elegant Graphics for Data Analysis*. New York, NY: Springer. Retrieved from https://ggplot2.tidyverse.org

Wooldridge, J. M. (2003). Cluster-sample methods in applied econometrics. *The American Economic Review*, *93*(2), 133–138. https://doi.org/10.1257/000282803321946930

Zeileis, A. (2004). Econometric computing with HC and HAC covariance matrix estimators. *Journal of Statistical Software, 11*(10), 1–17. https://doi.org/10.18637/jss.v011.i10

Zeileis, A. (2006). Object-oriented computation of sandwich estimators. *Journal of Statistical Software, 16*(9), 1–16. https://doi.org/10.18637/jss.v016.i09

Zeileis, A., & Hothorn, T. (2002). Diagnostic checking in regression relationships. *R News*, *2*(3), 7–10.
